# A multilevel school-based intervention on sedentary time in children: effectiveness and socioeconomic moderation results from the CIPRES cluster-RCT

**DOI:** 10.3389/fpubh.2026.1839833

**Published:** 2026-06-15

**Authors:** Steven Laujac, Olivier Gavarry, Sophie Lafond, Josselin Franceschi, Axel Rozand, Laurence Pallier, Cyrille Delpierre, Marion Carayol

**Affiliations:** 1 Youth - Physical and Sports Activity - Health Laboratory (JAP2S), University of Toulon, Toulon, France; 2 Centre Hospitalier Intercommunal Toulon-La Seyne sur Mer, Hôpital Sainte Musse, Toulon, France; 3 Comity of Health Education of Var Department (CODES 83), Toulon-La Garde, France; 4 EQUITY Team, CERPOP UMR 1295, Inserm-Université Toulouse III, Toulouse, France

**Keywords:** children, physical activity, school-based intervention, sedentary, socio-ecologic model, trans-contextual model, socioeconomic

## Abstract

**Background:**

Children’s sedentary behavior is a major public health priority. While school-based programs are common, research must explore more effective approaches using robust theoretical frameworks. The CIPRES cluster-randomized controlled trial evaluated a 6-week multi-component school-based intervention combining the Socio-Ecological perspective and Trans-Contextual Model to reduce children’s sedentary time (ST) and increase moderate-to-vigorous physical activity (MVPA).

**Methods:**

Four French municipalities were cluster-randomized to either the CIPRES or Control group. Participants were children in 3rd and 4th grades. The intervention was multilevel, using co-constructed activity booklets to target school, after-school, and home settings to reduce ST. Activities were driven by TCM principles. Device-based ST and MVPA were assessed via 7-day accelerometry (accelerometers GT3X+) at baseline and post-intervention. Intervention effects and the moderating role of the Ecological Deprivation Index were analyzed using multiple constrained linear mixed models on three distinct periods (entire week, after-school and non-school days).

**Results:**

A total of 543 children (49.2% girls; mean age 9.2 years; 76.2% highly deprived) participated. The intervention yielded significant group*time interaction effects over the whole week, preventing the increase in relative ST (*β* = −1.2 percentage points [pp], *p* = 0.033) and improving relative MVPA (*β* = +0.7 pp., *p* = 0.034) in the CIPRES group. These effects were primarily driven by the after-school period (relative ST: *β* = −2.5 pp., *p* = 0.002; relative MVPA: *β* = +1.4 pp., *p* = 0.003). No significant intervention effects were observed during non-school days. Crucially, deprivation level significantly moderated efficacy during the after-school period: the intervention significantly decreased ST and increased MVPA primarily among highly deprived participants, while slightly deprived participants exhibited behavioral deterioration.

**Conclusion:**

The theory-based CIPRES intervention effectively curbed the weekly accumulation of ST, driven by improvements during the after-school period. By predominantly benefiting highly deprived youth, it offers a promising strategy to reduce social health inequalities, though further strategies are required to effectively alter routines during non-school days.

## 1 Introduction

In an increasingly digitized society, sedentary behavior (SB), characterized by low energy expenditure while in a sitting or reclining posture (1), has become a major public health concern. In the long term, it is associated with an increased risk of all-cause mortality, the development of chronic diseases such as cancer, cardiovascular disease, and type 2 diabetes, and reduced quality of life (2–4). Among children and adolescents aged 5 to 17 years, sedentary time (ST) deserves particular attention. The main public health recommendation is to limit recreational screen time to no more than 2 h per day (5, 6). Exceeding this threshold is reportedly associated with poorer physical fitness, degraded cardiometabolic health, diminished mental health and quality of life, and a higher risk of depression (5, 7–9).

The scale of this phenomenon is alarming. In 2016, 81% of children and adolescents failed to meet the World Health Organization recommendations of at least 60 min of moderate-to-vigorous physical activity (MVPA) per day (10). This lack of physical activity (PA) is compounded by excessive screen exposure. In Europe, nearly 40% of individuals aged 6 to 9 years spend more than 2 h daily on screens (11). In France, this duration increases with age, rising from 3 h and 7 min among children aged 6–10 years to 5 h and 23 min among adolescents aged 15–17 years (12). This trend is confirmed by harmonized accelerometry data collected from 1999 to 2016 in 18 countries (mainly European), which revealed that youth (ages 2–18 years) spend, on average, almost half of their waking time sedentary (49.5%), compared with only 6.4% in MVPA (13). This trend is more pronounced in households with low socioeconomic status, which has been identified as an aggravating factor associated with SB (14–16). Nevertheless, few intervention studies targeting ST have examined the moderating effect of socioeconomic status on their outcomes (17, 18). Therefore, it is crucial to act as early as possible through multicomponent intervention strategies. In this context, the school setting represents a strategic environment for intervention, as it offers direct and prolonged access to many children living in the same socioeconomically deprived areas. However, recent meta-analyses on school-based interventions targeting ST report uncertain effectiveness, notably attributed to methodological discrepancies (19–23). Thus, research is shifting toward more robust theoretical approaches.

Multiple determinants of SB have been previously identified (24–26). The socio-ecological model (SEM) provides a useful framework for organizing these determinants into multiple interacting levels, such as individual, interpersonal, social, and environmental levels (27). Multilevel interventions guided by a socio-ecological approach have already been identified as a relevant strategy for targeting ST, particularly when strategies are deployed across four levels (28). Combining school, family, and broader environmental components has also been identified as a promising strategy (29, 30). In France, the ICAPS study demonstrated that a four-level intervention strategy, combining educational and behavioral components, social support from parents, teachers, and educators, and actions implemented in school, home, and community settings, can reduce television viewing and increase PA (31).

Previous studies have suggested that behavior change techniques may help target individual determinants (32, 33). Notably, theory-based interventions have already shown their effectiveness in reducing SB and promoting PA (28, 34). However, no consensus has yet been reached regarding the most effective strategy for achieving a sustained reduction in ST. The trans-contextual model (TCM) (35), integrating self-determination theory (36), the hierarchical model of intrinsic motivation (37), and the theory of planned behavior (38), suggests that teacher and parent support may foster autonomous motivation for PA and, in turn, behavioral intention across school and leisure contexts (39, 40). Although its effectiveness in increasing PA participation has been suggested (41–43), its potential for reducing children’s ST remains less well documented. Nevertheless, evidence associating theory of planned behavior constructs (attitude, subjective norms, and perceived behavioral control) with intention to remain sedentary (44–46), together with the potential protective effect of higher intrinsic and identified motivation on ST (47), suggests that the TCM is relevant for multilevel interventions targeting ST.

It is within this framework that the CIPRES intervention (*C*o-construct, *I*mplement and evaluate a multi-level intervention to *PRE*vent a sedentary lifestyle in children) is situated. Because ST is highly prevalent outside school hours (48), particularly within the family environment (49), the intervention was co-constructed through a participatory approach to extend beyond the school setting and account for social disparities. Its design relied on a dual theoretical anchoring, combining the SEM and the TCM in a hierarchically integrated and complementary manner. The socio-ecological perspective provides a macro-level framework that maps the different levels influencing behavior and identifies the key actors within each level, whereas the TCM offers a micro-level, for intervention contents elaboration.

The trial was implemented in the Var department (southern France), a heterogeneous territory of nearly 1.1 million inhabitants characterized by significant socio-demographic, geographic, and economic inequalities. It combines a densely urbanized 432 km coastal strip, where half the population resides, with a more rural inland area (50). The overall poverty rate is 16.8%, which is higher than the national average (14.5%). This deprivation particularly affects minors, with the Var displaying a poverty rate of 24% among those under 18 (compared to 22% nationally) (51). In 2024, the department counted 23 priority neighborhoods with a high concentration of low-income populations. Sixteen of these are grouped within just four municipalities targeted by the study: Toulon, the prefecture and core city of the Toulon Provence Méditerranée metropolis (10), La Seyne-sur-Mer (2) and Hyères (2), respectively the second and third largest municipalities in this metropolis, and Brignoles (2), the main town of the Provence Verte area. It is in this highly contrasted context, with an overall poverty rate ranging from 7.5% in Hyères to over 23% in Brignoles, that the CIPRES intervention was deployed (52). To address these health and social inequalities, local prevention policies are actively implemented across the territory, notably through the CODES 83 (Comité Départemental d’Éducation pour la Santé) and the “Maisons Sport-Santé,” supported by the Regional Health Agency (53, 54).

This article presents the results of a cluster-randomized controlled trial designed to evaluate the effect of the CIPRES intervention on ST among children in 3rd and 4th grades. The intervention was a school-based multilevel program incorporating theory-based health promotion components targeting ST. The study hypotheses were that the CIPRES intervention would significantly reduce children’s ST, which was the primary outcome, and increase MVPA, which was the secondary outcome. The secondary objective was to evaluate whether background deprivation moderated the effectiveness of the intervention.

## 2 Methods

This study was part of the CIPRES project. The detailed protocol has been previously published (55).

### 2.1 Study design

The CIPRES study employed a two-arm, prospective, cluster-randomized controlled trial design, that was previously detailed (55). The trial was conducted in four communities from the Var Department, southern France (Toulon, La Seyne-Sur-Mer, Hyères, Brignoles). To prevent contamination between groups, cluster randomization was performed at the municipality level. La Seyne-Sur-Mer and Hyères were assigned to the control group (no intervention), whereas Toulon and Brignoles formed the CIPRES group (received the intervention). Randomization was carried out through Stata software (Version 16, StataCorp LLC, College Station, TX). The intervention was implemented during the 2021–22 school year (September 2021–July 2022). This trial was reported in accordance with the CONSORT 2010 statement (see Supplementary File 1). A detailed description of the intervention is provided in accordance with the TIDieR checklist (see Supplementary File 2).

### 2.2 Participants

The study population comprised children enrolled in participating primary schools in the selected municipalities. Eligibility criteria were as follows: children had to be enrolled in a participating primary school, be in 3rd and 4th grades, and, owing to the French system allowing multi-grade classes, 5th grade pupils in 4th/5th grade classes were also eligible. In addition, written informed consent had to be obtained from the parents. Children were excluded if they explicitly refused to take part or if their characteristics did not allow accelerometer-based assessment. Before baseline, teachers distributed the consent forms to pupils, who took them home for their parents to complete and return. The information provided to parents explained the study aims and assessment procedures, including the use of self-administered questionnaires and accelerometers worn by children for 7 days. Additionally, the children provided oral assents. In accordance with the Declaration of Helsinki, written ethical approval was obtained from the ethics evaluation committee of Inserm, the Institutional Review Board (IRB00003888, IORG0003254, FWA00005831) of the French Institute of Medical Research and Health.

### 2.3 Intervention

The CIPRES intervention aimed to address multiple levels to reduce children’s ST: (i) intrapersonal (children), (ii) interpersonal (parents, teachers, educators), (iii) organizational (home and school environments), and (iv) community (municipality, local associations). Unfortunately, the COVID-19 pandemic precluded community-level intervention. Nevertheless, the three other levels were targeted in accordance with the protocol. Given that meta-analysis reported that school-based intervention duration may not be the first determinant of effectiveness (56), feasibility was prioritized. Accordingly, the CIPRES intervention was implemented over a six-week period, nested between two school holiday breaks. The intervention group participated in multiple activities across various settings involving teachers, parents, and educators. A full description of the intervention content is available in the protocol publication (55).

Briefly, teachers were provided with a booklet containing six ready-to-use classroom activities linked to specific school subjects, such as French, mathematics, physical education, and moral and civic education. Each activity aimed at raising children’s awareness of SB and reducing ST while targeted one or more constructs of the TCM (autonomous motivation, attitude, subjective norms, perceived behavioral control or intention). For example, these activities included defining terms related to PA and SB in French classes; converting units of time to create an activity schedule in mathematics; engaging in PA while identifying the concepts of sedentary vs. PA in physical education; and autonomously developing alternatives to SB in moral and civics education. A supplementary booklet offered a wider choice of activities to adapt the intervention to the children’s specific characteristics.

The extracurricular booklet, designed for educators working with children during the after-school periods, aimed to reinforce the school-based messages by offering seven practical activities, such as debate games, screen-time substitution tasks, the “10,000 Step” challenge, “Fitness trail,” and poster design. These activities were explicitly framed to increase awareness of SB and to maintain autonomy-supportive guidance outside school hours.

Finally, the family booklet, intended for parents and children, provided scientific information on SB, screen use, PA, and their health consequences, together with recommendations, practical advice, family activities, digital tools, and web resources to support healthier routines at home, encourage alternatives to screen-based ST and parental autonomy support.

### 2.4 Evaluations

The CIPRES intervention’s effect on the primary outcome (ST) and secondary outcome (MVPA) was evaluated by comparing the CIPRES group to the control group at baseline (T0, pre-intervention) and 6 weeks post-intervention (T1). All assessments were conducted within the schools on a class-by-class basis during regular school hours. Considering potential seasonal influences on activity levels (57), the evaluation schedule was distributed evenly across groups throughout the year to minimize bias.

#### 2.4.1 Device-based measurement

Children’s ST and MVPA were monitored for seven consecutive days through a triaxial ActiGraph GT3X + accelerometer (ActiGraph, Pensacola, FL, USA). To increase comfort and compliance, the device was worn on the non-dominant wrist, and participants were instructed to remove it only for water-based activities. The data were sampled at 60 Hz and processed via ActiLife software (v6.13.4). Non-wear time was identified via the Choi et al. algorithm (58), defined as ≥90 consecutive minutes of zero counts, allowing for a 2-min spike tolerance within a 30-min window. The data were aggregated into 5-s epochs. For inclusion in the analysis, participants were required to have a minimum amount of valid wear time (VWT). It was calculated by subtracting the non-wear time from the total monitored time. To improve interpretability, the analysis focused on three distinct periods of children’s lives. The specific VWT criterion was at least four valid days, including one weekend day, with each valid day containing ≥600 min of VWT between 7:00 a.m. and 10:00 p.m. for Period 1 (Entire Week). A minimum VWT of 1,080 min (Aggregated over 3 days) was required across weekends and Wednesdays (7:00 a.m.–10:00 p.m.) for Period 2 (Non-School Days). Finally, a minimum VWT of 528 min (Aggregated over 4 days) was required during schooldays between 4:30 p.m. and 10:00 p.m. for Period 3 (after-school). Then, ST and MVPA were quantified using the validated Crouter et al. vertical axis cut-points (59) (ST ≤ 35 counts/5 s and MVPA ≥361 counts/5 s). Outcomes are reported as absolute minutes and as a percentage of VWT.

#### 2.4.2 Demographic characteristics

A self-administered questionnaire, previously detailed in the protocol paper (55), was administered to parents at each time point. This questionnaire consists of several items regarding children’s behavior; it might be noticed that only weight and height were used in the present article. The body mass index (BMI) was then calculated (kg/m^2^). The children’s age was calculated from their dates of birth, which were obtained from schools. Each participating school was categorized by socioeconomic deprivation level, which was based on geographic location. The French Ecological Deprivation Index (EDI) characterizes deprivation at the most granular French geographical level, incorporating objective and subjective poverty indicators and fundamental needs indicators (60). The EDI is then divided into quintiles (from Q1 to Q2 = slightly deprived, and from Q3 to Q5 = highly deprived).

#### 2.4.3 Fidelity

Before the intervention began, information meetings were organized between the research team, teachers, and extracurricular educators to present the intervention materials. Intervention fidelity was assessed by the research team. Regular contact with the teachers allowed monitoring of the activities implementation as the intervention progressed. Additionally, each teacher participating in the study was required to record the activities implemented in class within a table provided with the activity booklet. Teachers were encouraged to write the adaptations provided to implemented activities. Regular contact was also maintained with the extracurricular educators and the research team to ensure that they used the provided activities. However, we were unable to collect quantitative indicators from the educators and families, which made it impossible to report.

### 2.5 Statistical analysis

Descriptive statistics were calculated for the anthropometric variables, ST, MVPA, school level, and EDI, and are reported as the means (SDs) or frequency (%). Baseline differences between the CIPRES and control groups were assessed through independent sample t-tests (or nonparametric alternatives) for quantitative variables and chi-square tests for categorical variables. To compare differences over time between groups while accounting for repeated measures and missing data under the intent-to-treat principle, linear mixed models (LMM) utilizing a Constrained Longitudinal Data Analysis approach were employed. Parameterization includes the baseline measurement as part of the response vector and mathematically constrains the baseline means to be equal between the randomized groups (61). Time was modeled as a categorical fixed effect to evaluate treatment differences at post-intervention. Fixed effects included time and time-by-group interactions along with age, sex, and the EDI as covariates. To account for the hierarchical structure of the data, the models incorporated nested random effects. A maximal random effect structure (i.e., participants nested within classes, schools and municipalities) was initially applied. The magnitude of the effect was quantified using a standardized Cohen’s d effect size, with values of 0.2, 0.5, and 0.8 interpreted as small, medium, and large effects, respectively (62). For variables where singular fits were detected, the random structure was parsimoniously simplified by removing the level(s) with zero variance to ensure model stability (63). The assumptions for each test were verified, including the variance inflation factor (VIF < 5). *Post hoc* comparisons of estimated marginal means were conducted to specify within-group treatment effects at post-intervention. The EDI moderator effect was assessed with a step-down analytical approach. First, a three-way interaction term (Time*Group* EDI) was introduced into the primary LMM to test whether the intervention effect significantly differed across EDI categories. Secondary analyses were then conducted only for variables whereas the three-way interaction was statistically significant. For these specific variables, a secondary analysis was performed exclusively within the CIPRES group. In this within-group model, Time*EDI interaction terms were used to evaluate how changes in MVPA and ST varied across the different EDI categories, with the ‘slightly deprived’ category serving as the reference group. All tests were two-sided with *α* = 0.05. Analyses were conducted via R software (Version 4.4.2).

## 3 Results

### 3.1 Participant characteristics

**Figure 1:**
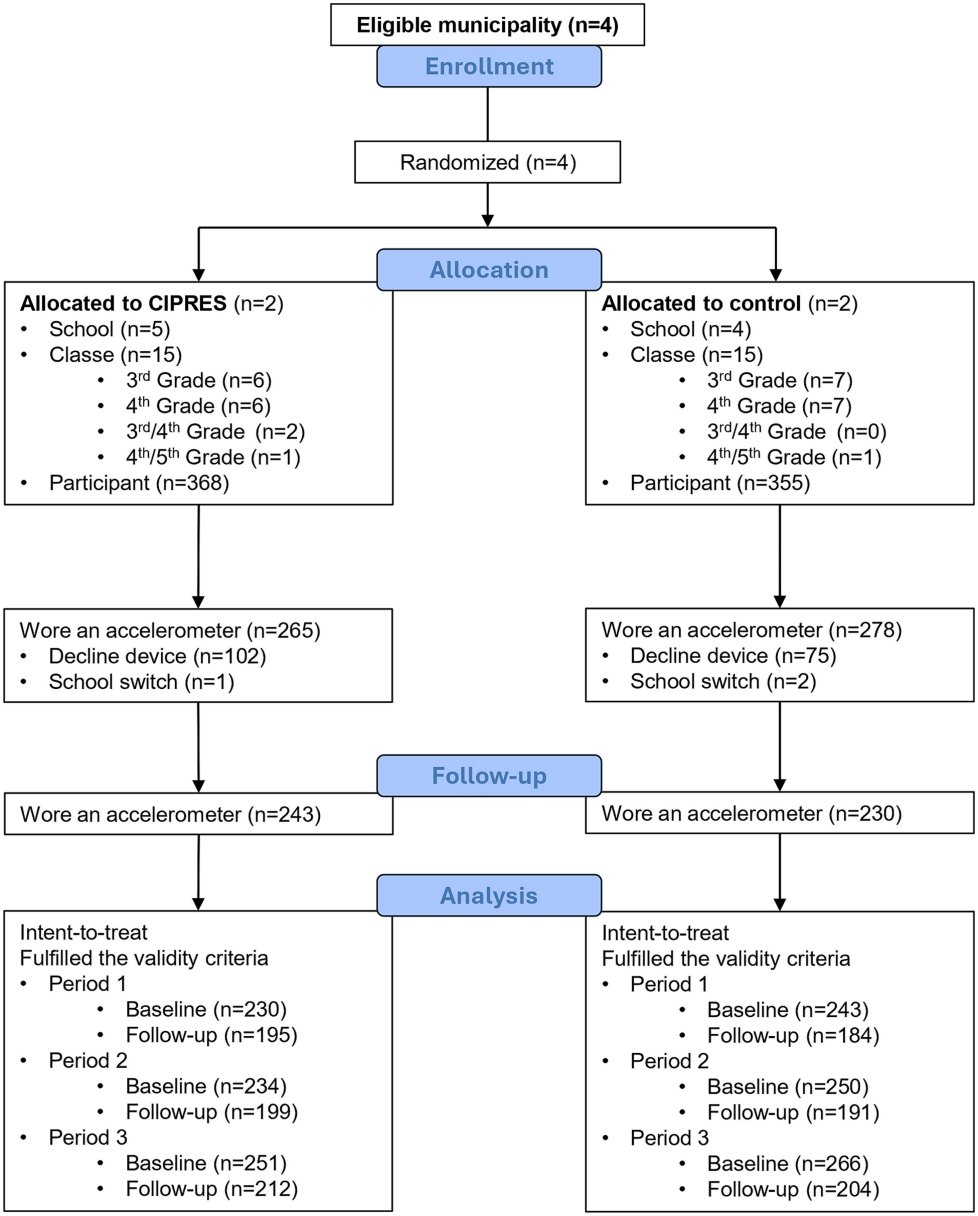
Flow diagram of the inclusion process.

The intervention was implemented between September 2021 and July 2022 in 9 schools located in four cities that were randomly assigned to either the CIPRES or the control group. A total of 30 teachers from these schools participated in the study, drawn from 3rd Grade (*n* = 13), 4th Grade (*n* = 13), and multi-grade classrooms (3rd/4th Grade, *n* = 2; 4th/5th Grade, *n* = 2). This resulted in 15 teachers in the CIPRES group and 15 in the control group. Out of a target sample of 723 children, a total of 543 (49.2% girls) consented to participate in the study (Figure 1). In the control group, participants came from La Seyne-sur-Mer (84.5%) and Hyères (15.5%), whereas the CIPRES group included participants from Brignoles (52.1%) and Toulon (47.9%).

The baseline characteristics of the participants are reported in Table 1. The total sample had a mean age of 9.2 years (SD = 0.7) and a mean BMI of 17.1 kg/m^2^ (SD = 2.8) and most participants were recruited from areas of high socioeconomic deprivation (76.2%). Participants spent 45.4% of their weekly time in ST versus 18.1% in MVPA. Baseline comparisons revealed that participants in the CIPRES group were significantly older (+0.3 years, *p* < 0.001), taller (+1.8 cm, *p* = 0.033), and heavier (+1.5 kg, *p* = 0.028) than those in the control group, but their BMI were similar (+0.2 kg/m^2^, *p* = 0.646) (Table 1). The CIPRES group was, however, significantly less socioeconomically deprived (*p* < 0.001). A total of 473, 484, and 517 children met the minimum wear-time criteria for valid accelerometry data in Periods 1, 2, and 3, respectively. Importantly, baseline levels of ST and MVPA did not differ significantly between the groups in any of these periods.

**Table 1:** Baseline characteristics of the enrolled children.

Variable	Missing data *N*	Total sample Mean (SD)/%	Control Mean (SD)/%	CIPRES Mean (SD)/%
		*N* = 543	*N* = 278	*N* = 265
Demographic characteristics				
Girls		49.2	50.4	47.9
Age (years)^#^		9.2 (0.7)	9.0 (0.7)	9.3 (0.7)
Height (cm)^#^	179	135.4 (7.7)	134.5 (7.1)	136.3 (8.1)
Weight (kg)^#^	160	31.7 (6.9)	30.9 (6.7)	32.4 (7.0)
BMI (kg/m^2^)	187	17.1 (2.8)	17.0 (2.6)	17.2 (2.9)
Educational level				
3rd Grade		46.6	46.4	46.8
4th Grade		50.5	49.3	51.7
5th Grade		2.9	4.3	1.5
EDI level^#^				
Slightly deprived		23.8	15.5	32.5
Highly deprived		76.2	84.5	67.5
Sedentary/physical activity levels				
*Period 1*		*N* = 473	*N* = 243	*N* = 230
Absolute ST (min/week)		2,647.1 (467.3)	2,617.0 (453.1)	2,679.0 (480.8)
Relative ST (%)		45.4 (5.9)	45.2 (5.7)	45.6 (6.1)
Absolute MVPA (min/week)		1,053.3 (241.1)	1,057.5 (232.5)	1,048.8 (250.3)
Relative MVPA (%)		18.1 (3.7)	18.3 (3.5)	17.9 (3.9)
VWT (min)		5,825.2 (633.2)	5,790.1 (653.8)	5,862.3 (609.9)
*Period 2*		*N* = 484	*N* = 250	*N* = 234
Absolute ST (min/week)		1264.1 (266.6)	1257.7 (260.0)	1270.8 (274.0)
Relative ST (%)		50.9 (8.4)	51.1 (8.0)	50.7 (8.7)
Absolute MVPA (min/week)		400.9 (129.1)	392.2 (122.2)	410.2 (135.7)
Relative MVPA (%)		16.2 (4.8)	15.9 (4.5)	16.4 (5.0)
VWT (min)		2482.4 (334.9)	2463.4 (354.6)	2502.8 (312.0)
*Period 3*		*N* = 517	*N* = 266	*N* = 251
Absolute ST (min/week)		603.6 (140.8)	597.3 (139.4)	610.3 (142.3)
Relative ST (%)		49.7 (9.1)	49.3 (9.0)	50.1 (9.3)
Absolute MVPA (min/week)		208.2 (70.2)	209.9 (69.2)	206.4 (71.4)
Relative MVPA (%)		17.1 (5.2)	17.4 (5.3)	16.9 (5.1)
VWT (min)		1214.7 (178.1)	1210.0 (175.7)	1219.6 (180.8)

Period 1: Entire week between 7:00 a.m. and 10:00 p.m. Period 2: without school days between 7:00 a.m. and 10:00 p.m. Period 3: school days, between 4:30 to 10:00 p.m. Slightly deprived: EDI Q1 to Q2. Highly deprived: EDI Q3–Q5. EDI, ecological deprivation index; VWT, valid wear time; ST, sedentary time; MVPA, moderate to vigorous physical activity; ^#^Baseline significant difference between-group (*p* < 0.05).

### 3.2 Intervention fidelity

Intervention fidelity data revealed the successful delivery of the school-based component, with participants receiving a median of 5 sessions [IQR: 4–7] out of the 6 planned. However, implementation varied from one to eight sessions, highlighted by two extreme cases in the municipality of Toulon. In one class, only a single session was conducted due to scheduling constraints, while eight sessions were implemented in another class at a different school. Despite this variability, all activities included in the educational booklet were utilized across the participating schools. The activities implemented by the teachers were part of the main booklet, and none of the activities from the supplementary booklet were used. The family booklets were successfully distributed. All children in the CIPRES group received a copy intended for home use with their parents. Concurrently, after-school educators responded favorably to the activity booklet. However, we were unable to collect sufficiently robust quantitative metrics regarding the actual utilization of these materials in both the family and after-school settings. As a direct result of COVID-19 restrictions, the community-level component of the intervention could not be carried out.

### 3.3 Accelerometer-based assessment

Analysis of the VWT showed no significant group-by-time interaction effect for any period (all *p* > 0.14), suggesting that the interaction effect on ST and MVPA was not due to systematic variations in wear time between groups.

#### 3.3.1 Effects of the CIPRES intervention on sedentary time

**Figure 2:**
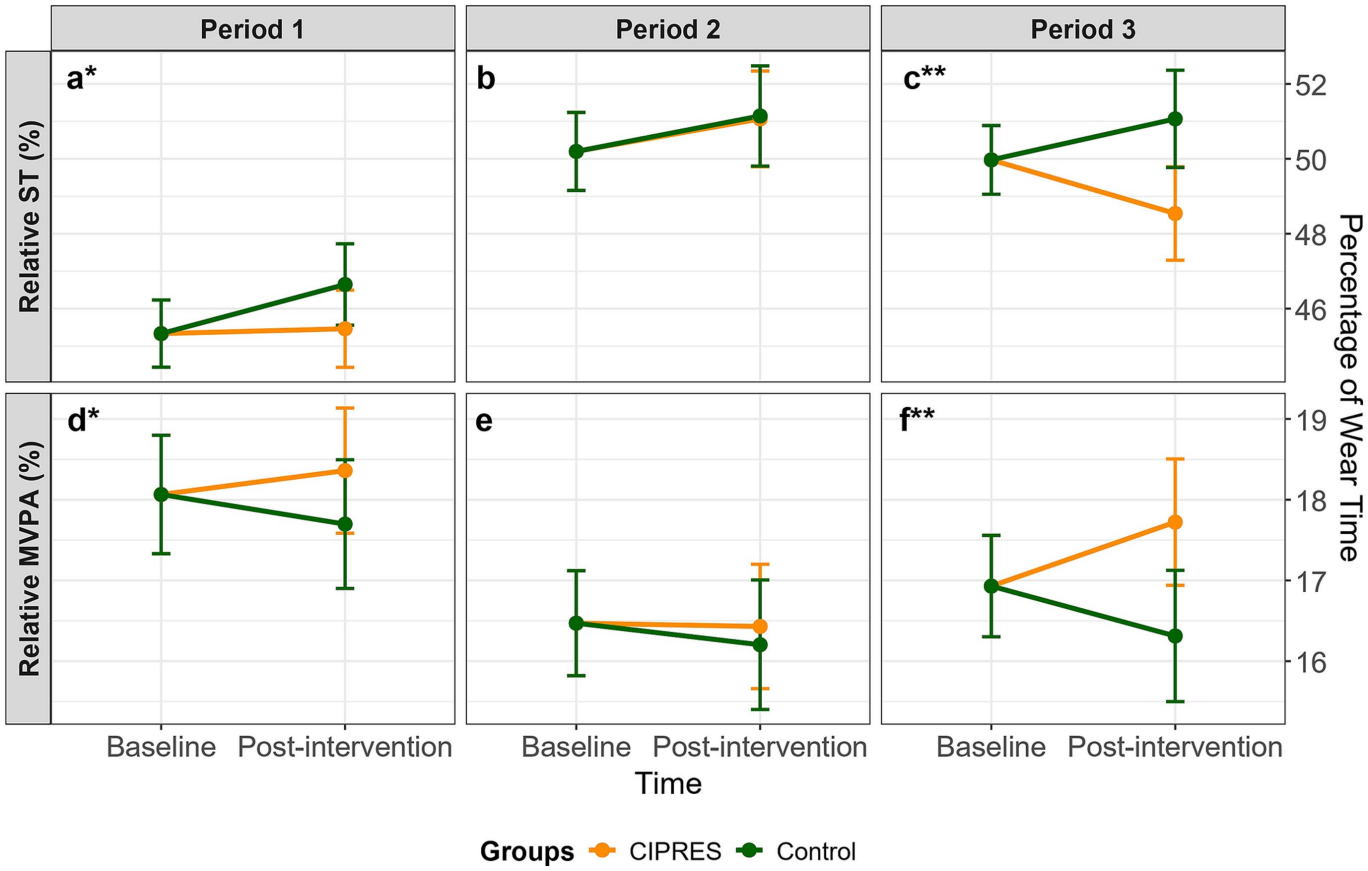
Changes over time in ST and MVPA, broken down by group and period. **(a–c)** Show changes in the relative STs (%) by group for periods 1, 2 and 3, respectively. **(d–f)** Show changes in MVPA (%) by group for periods 1, 2 and 3, respectively. The results are presented as the means (± standard errors). Significant Group-by-Time interaction effect: *p* value, * < 0.05, ** < 0.01. P1: Period 1, the whole week (7:00 a.m.–10:00 p.m.); P2: Period 2, non-school days (7:00 a.m.–10:00 p.m.); P3: Period 3, after-school period (4:30–10:00 p.m.).

Period 1: analysis revealed a statistically significant group*time interaction effect for absolute (*β* = −113.5 min, *p* = 0.024, *d* = −0.22), and relative ST (*β* = −1.2 pp., *p* = 0.033, *d* = −0.19) (Table 2; Figure 2a). Within-group analyses showed that both the CIPRES and control groups experienced significant decreases in absolute ST over time (Estimated Difference [ED] = −227.2 min, *p* < 0.001 and ED = −113.7 min, *p* = 0.003, respectively). Regarding relative ST, the interaction was driven by a significant increase in the control group (ED = 1.3 pp., *p* = 0.002), whereas the CIPRES group remained stable (ED = 0.1 pp., *p* = 0.749).

**Table 2:** Estimated marginal means and intervention effects on post-intervention using a constrained linear mixed model.

Variable	Groupe	EMM (SE)	Coefficient *β* (SE)	[95% CI]	*p*-value	Cohen’s *d*
Period 1: Entire week between 7:00 a.m. and 10:00 p.m.						
Relative ST (%)	Control	46.6 (0.5)	Réf.			
	CIPRES	45.5 (0.5)	**−1.2 (0.6)**	**[−2.3; −0.1]**	0.033*	**−0.19**
Absolute ST (min)	Control	2543.5 (41.6)	Réf.			
	CIPRES	2430.0 (39.5)	**−113.5 (50.0)**	**[−211.6; −15.4]**	0.024*	**−0.22**
Relative MVPA (%)	Control	17.7 (0.4)	Réf.			
	CIPRES	18.4 (0.4)	**0.7 (0.3)**	**[0.1; 1.3]**	0.034*	**0.17**
Absolute MVPA (min)	Control	973.8 (31.2)	Réf.			
	CIPRES	984.6 (31.0)	10.9 (24.0)	[−36.1; 57.8]	0.663	0.04
Period 2: without school days between 7:00 a.m. and 10:00 p.m.						
Relative ST (%)	Control	51.1 (0.7)	Réf.			
	CIPRES	51.1 (0.6)	−0.1 (0.8)	[−1.6; 1.4]	0.916	−0.01
Absolute ST (min)	Control	1180.5 (21.7)	Réf.			
	CIPRES	1184.7 (20.7)	4.2 (26.5)	[−48.7; 56.1]	0.875	0.02
Relative MVPA (%)	Control	16.2 (0.4)	Réf.			
	CIPRES	16.4 (0.4)	0.2 (0.4)	[−0.6; 1.1]	0.594	0.05
Absolute MVPA (min)	Control	379.7 (11.6)	Réf.			
	CIPRES	384.6 (11.0)	5.0 (12.4)	[−19.2; 29.2]	0.688	0.04
Period 3: school days, between 4:30 to 10:00 p.m.						
Relative ST (%)	Control	51.0 (0.7)	Réf.			
	CIPRES	48.5 (0.6)	**−2.5 (0.8)**	**[−4.2; −0.9]**	0.002**	**−0.27**
Absolute ST (min)	Control	577.7 (10.7)	Réf.			
	CIPRES	537.6 (10.2)	**−40.1 (13.5)**	**[−66.6; −13.6]**	0.004**	**−0.28**
Relative MVPA (%)	Control	16.3 (0.4)	Réf.			
	CIPRES	17.7 (0.4)	**1.4 (0.5)**	**[0.5; 2.3]**	0.003**	**0.26**
Absolute MVPA (min)	Control	183.1 (5.8)	Réf.			
	CIPRES	194.6 (5.6)	11.5 (6.2)	[−0.6; 23.6]	0.065	0.16

EMM, estimated marginal mean; SE, standard error; CI, confidence interval. EMMs, treatment differences, 95% CIs, and *p*-values were estimated using a linear mixed models applying a constrained baseline approach. The model included time and time-by-group interaction as fixed effect, and age, gender, and EDI category as covariates. A random intercept for Commune/School/Class/Subject was included to account for hierarchical data clustering. The treatment difference represents the CIPRES group minus the Control group at post-intervention. *p < 0.05, **p < 0.01. Significant results are indicated in bold.

Period 2: no significant interaction effect was detected on absolute and relative ST during non-school days (all *p* > 0.88) (Table 2; Figure 2b).

Period 3: analysis demonstrated a statistically significant group*time interaction effect for absolute (*β* = −40.1 min, *p* = 0.003, *d* = −0.28), and relative ST (*β* = −2.5 pp., *p* = 0.002, *d* = −0.27) (Table 2; Figure 2c). Within-group analyses demonstrated that the interaction for absolute ST was driven by a decrease in the CIPRES group (ED = −68.5 min, *p* < 0.001) and the control group (ED = −28.4 min, *p* = 0.008). For relative ST, the CIPRES group exhibited a significant decrease (ED = −1.4 pp., *p* = 0.022), while the control group showed a non-significant increase (ED = +1.1 pp., *p* = 0.085).

#### 3.3.2 Effects of the CIPRES intervention on moderate to vigorous physical activity

Period 1: analysis showed no statistically significant group*time interaction effect for absolute MVPA (*β* = 10.9 min, *p* = 0.651, *d* = 0.04). However, a significant interaction was observed for relative MVPA (*β* = 0.7 pp., *p* = 0.034, *d* = 0.17) (Table 2; Figure 2). Within-group analyses for relative MVPA showed that neither the CIPRES group (ED = 0.3 pp., *p* = 0.183) nor the control group (ED = −0.4 pp., *p* = 0.110) experienced statistically significant changes.

Period 2: no intervention effect was observed on absolute and relative MVPA during non-school days (all *p* > 0.59) (Table 2; Figure 2e).

Period 3: analysis showed no statistically significant group*time interaction effect for absolute MVPA (*β* = 11.5 min, *p* = 0.063, *d* = 0.16). However, a significant interaction was observed for relative MVPA (*β* = 1.4 pp., *p* = 0.003, *d* = 0.26) (Table 2; Figure 2f). Within-group analyses showed that for relative MVPA, the interaction was driven by a significant increase in the CIPRES group (ED = 0.8 pp., *p* = 0.021), compared to a non-significant decrease in the control group (ED = −0.6 pp., *p* = 0.077).

#### 3.3.3 Ecological deprivation index moderator effect

Moderation analyses were conducted to determine whether the intervention’s efficacy varied according to level of deprivation. Analysis revealed a statistically significant effect of EDI within a three-way interaction (Time*Group*EDI) during *Period 3* for absolute (*β* = −83.0 min, *p* = 0.011) and relative ST (*β* = −8.0 pp., *p* < 0.001), as well as for relative MVPA (*β* = 3.8 pp., *p* < 0.001).

Within the CIPRES group, a two-way interaction analysis (Time*EDI) showed a statistically significant effect of EDI on *Period 3* for both absolute (*β* = −104.5 min, *p* < 0.001, *d* = −0.71) and relative ST (*β* = −6.2 pp., *p* < 0.001, *d* = −0.65). Within-group analyses revealed that children from *highly deprived* schools experienced significant decreases in both absolute (ED = −101.1 min, *p* < 0.001) and relative ST (ED = −3.3 pp., *p* < 0.001). Conversely, children from *slightly deprived* schools showed no significant change in absolute ST (ED = 3.47 min, *p* = 0.862) and a statistically significant increase in relative ST (ED = 2.87 pp., *p* = 0.013). Additionally, a statistically significant interaction effect was found for relative MVPA (*β* = 2.5 pp., *p* < 0.001, *d* = 0.45). This was driven by a significant increase in relative MVPA specifically within children from *highly deprived* schools (ED = 1.6 pp., *p* < 0.001), while children from *slightly deprived* schools experienced a non-significant decrease (ED = −0.9 pp., *p* = 0.142). Results are summarized in Table 3.

**Table 3:** Estimated marginal means and intervention effects at post-intervention regarding EDI for CIPRES group using a constrained linear mixed model during Period 3.

Variable/Groupe	EMM (SE)	Coefficient β (SE)	[95% CI]	*p*-value	Cohen’s *d*
Period 3: school days, between 4:30 to 10:00 p.m.					
Relative ST (%)					
Slightly deprived	53.0 (1.1)	Réf.			
Highly deprived	46.8 (0.7)	**−6.2 (1.3)**	**[−8.7; −3.6]**	**<0.001**	**−0.65**
Absolute ST (min)					
Slightly deprived	615.6 (20.3)	Réf.			
Highly deprived	511.1 (14.0)	**−104.5 (23.1)**	**[−148.6; −60.4]**	**<0.001**	**−0.71**
Relative MVPA (%)					
Slightly deprived	16.0 (0.6)	Réf.			
Highly deprived	18.4 (0.4)	**2.5 (0.7)**	**[1.1; 3.8]**	**<0.001**	**0.45**
Absolute MVPA (min)					
Slightly deprived	185.4 (8.4)	Réf.			
Highly deprived	199.9 (5.9)	14.5 (9.4)	[−3.5; 32.5]	0.124	0.20

EMM, estimated marginal mean; SE, standard error; CI, confidence interval. EMMs, treatment differences, 95% CIs, and *p*-values were estimated using a linear mixed models applying a constrained baseline approach. The model included time (Visit), the time-by-EDI interaction, age and gender as fixed effects on Period 3 for CIPRES Group. The difference represents the highly deprived group minus the slightly group at post-intervention. Significant results are indicated in bold.

## 4 Discussion

This study evaluated the effectiveness of the CIPRES intervention. The study demonstrated the feasibility of implementing a multilevel intervention across multiple schools in diverse cities, engaging teachers, parents, and educators with children from various socioeconomic backgrounds. The intervention had a protective effect on SB over the entire week, driven by a significant reduction on relative and absolute STs during the after-school period. This represents a highly significant finding, given the well-documented negative impact of SB on health. Concurrently, MVPA increased throughout the week, with a more pronounced effect after school. Additionally, the results revealed a moderator effect of the deprivation level on the intervention effect, with a better efficacy of intervention in participants from highly deprived schools. In contrast, an informative finding is the absence of a significant intervention effect during non-school days.

The intervention’s success during the after-school period (4:30 p.m.–10:00 p.m.) validates our program’s targeted approach. One of the theoretical frameworks guiding this intervention is characterized by its focus on individual determinants of behavior change within the school setting, aiming to facilitate the transfer of these changes to periods of unstructured leisure time (39). While the TCM has demonstrated effectiveness in increasing PA levels (42, 43), the present findings may suggest its potential utility for reducing SB. However, as the core constructs of the TCM have not been measured (i.e., autonomous motivation, attitudes, subjective norms, perceived behavioral control and intentions), no definitive conclusions regarding its efficacy can be drawn. The after-school period, which is particularly conducive to SB (48), might be influenced by intervention components specifically designed for this period, notably the booklets for home and after-school educators. The observed effectiveness is also potentially due to teachers raising awareness of ST-related issues during school hours. Furthermore, the activities in the teacher’s booklet, which offered alternatives to ST, may have had a direct consequence. By providing engaging and educational alternatives, these activities likely enabled a reallocation of time, in which periods of ST were replaced with PA. This direct substitution would explain both the significant decrease in relative ST (−1.4 pp) and the concurrent increase in relative MVPA (+0.8 pp) in the CIPRES group during the after-school period. These results support findings from previous school-based interventions showing similar efficacy in reducing after-school ST and increasing MVPA within school-based interventions (64–66).

Crucially, this localized effect continued throughout the entire week. The intervention helped stabilize ST and preserve MVPA levels in the CIPRES group, whereas the control group experienced a negative trend in these behaviors (increased ST and decreased MVPA). Therefore, the intervention had a notable preventive effect, as it protected children from the negative trajectory observed in the control group. This result is particularly compelling, given the tendency for ST to increase with age (12, 13) and the principle that habits established in childhood favor future behaviors (67). Consequently, preventing the increase in SB from a young age could yield substantial long-term public health benefits. Nevertheless, in the absence of long-term measurements, these findings should be interpreted with caution.

The absence of a non-school days intervention effect aligns with previous findings (68, 69) and highlights the contextual limitations of the intervention. The use of three distinct measurement periods allowed for a nuanced analysis. Consistent with previous studies (49, 70, 71), participants in this study presented greater relative ST (+5.5 pp) and lower relative MVPA (−1.9 pp) during non-school days than throughout the entire week at baseline. Therefore, two main hypotheses can explain the lack of intervention effects during non-school days. First, the influence of our program, which is strongly rooted in the school environment, could diminish as children move away from that setting. Second, weekend habits could be more heavily determined by the family context and, notably, by parents’ levels of ST and PA (49, 72, 73). Although a strategy centered on the family context has already shown its effectiveness (74), the booklet to parents alone seemed insufficient to alter well-established family routines, in which screen time could be predominant (49, 75). This point is particularly relevant, as our population, drawn mainly from socioeconomically disadvantaged backgrounds, would be more likely to exhibit higher levels of ST (14–16). We cannot exclude the possibility that the intervention’s effect was attenuated by parental habits that were only partially targeted by our intervention. This is particularly relevant given the strong association between parental and child SB (49), making these routines especially resistant to change in disadvantaged settings where screen time is already greater (76). Therefore, targeting a reduction in children’s ST during weekends may also require changing parents’ behavior patterns.

Another major finding of this study is the moderating effect of deprivation level on intervention efficacy. Indeed, the CIPRES intervention appeared to provide greater benefits to participants from highly deprived schools during the after-school period, driving a decrease in ST and an increase in MVPA. This is a particularly noteworthy finding given the strong predisposition of disadvantaged populations to engage in deleterious health behaviors, such as excessive screen time and other SB, compared to their more advantaged counterparts (14–16). Paradoxically, participants from slightly deprived schools appeared to exhibit behavioral deterioration, increasing their ST and decreasing their MVPA over this same period, a trend we are unable to explain at this stage. We hypothesized that, contrary to the Inverse Equity Hypothesis (77), which posits that novel public health interventions initially exacerbate social health inequalities by primarily benefiting advantaged populations, the CIPRES intervention might help mitigate these disparities by primarily reaching vulnerable populations. This may be attributed to a design that more effectively tailored the intervention to the needs of highly deprived schools, or alternatively, to a school-level effect. Nevertheless, further investigations are needed to elucidate the underlying mechanisms driving the deterioration of ST and MVPA levels among the most advantaged populations.

Finally, results show that in some cases, the changes in ST and MVPA are directionally opposite when absolute values are compared with relative values. This observation may be explained by the influence of VWT, as its impact on the assessment of ST and MVPA has been previously demonstrated (78). Thus, although the group-by-time interaction effect on VWT was not significant for any period (all *p* > 0.08), the VWT may have decreased between baseline and follow-up in both groups. This decrease mechanically led to lower absolute ST and MVPA levels, even if they increased in relative terms. Therefore, we postulate that verifying and quantifying VWT is critical for the interpretation of the results and that relative ST and MVPA should be prioritized in the analysis over absolute counterparts. Nevertheless, the positive effect of the intervention on absolute ST is corroborated by the relative values. The group comparison revealed that after 6 weeks, the intervention made it possible to reduce weekly ST by 109 min. This represents a highly significant finding, given the negative impact of SB on health.

### 4.1 Limitations

Several limitations must be considered. First, the fourth level of the SEM (community) could not be implemented due to pandemic-related restrictions, which may have limited the overall scope of the intervention. Second, the assessment process itself (wearing an accelerometer) may have influenced the behavior of the control group, potentially reducing the observed contrast between groups (assessment reactivity). Third, the study lacks long-term follow-up data, which prevents conclusions about the sustainability of the observed behavioral changes. Fourth, the disparity in sample size between deprivation level groups suggests that the findings regarding moderation effect of deprivation should be interpreted with caution. Finally, although implementation of teacher interventional activities was satisfactory, fidelity assessment lacked quantitative indicators regarding the use of family and extracurricular activities.

### 4.2 Strengths

The primary strength and originality of the CIPRES study lies in its dual theory driven framework, an innovative approach to reducing children’s SB outside of school. The use of a participatory co-construction methodology with local stakeholders aimed to improve the feasibility and implementation of the program. Another strength is the device-based assessment with nuanced methodology, which uses accelerometers to differentiate behavioral patterns across three distinct time periods (whole week, non-school days, and after-school). Additionally, the randomized controlled trial design and the robustness of the statistical analyses (constrained mixed linear models) reinforce the internal validity of the results. Furthermore, this study achieved a high retention rate (75%) despite COVID-19 challenges. These rates compare favorably with a meta-analysis reporting 80% recruitment in European school-based PA interventions (79), suggesting good engagement and representativeness within this context. This is particularly encouraging given the documented negative impacts of pandemic lockdowns on PA and SB (80).

### 4.3 Future research directions

From a practical standpoint, our results suggest that targeting the after-school period is a high-potential strategy for effective interventions aimed at reducing child SB, with simple tools such as activity booklets for educational staff serving as promising levers for change. To build upon these findings, future research should prioritize several key avenues. The primary challenge remains the design and evaluation of interventions, possibly involving more active parental strategies, that can effectively extend these benefits to the weekend. Further investigation is needed to determine the optimal dose and duration of the intervention for long-term sustainability and to ascertain whether the observed protective effect translates into tangible health outcomes. Finally, evaluating the enhanced efficacy of an intervention that also engages additional levels of the SEM, such as community-wide initiatives, represents a critical next step toward developing more holistic and impactful programs.

## 5 Conclusion

The findings suggest that the CIPRES intervention, which leverages a framework combining the SEM and TCM, induced favorable short-term changes in ST and MVPA in children. The intervention led to a relative improvement in ST (preventing the increase observed in controls) and MVPA over the week, driven primarily by significant positive changes during the after-school period. However, the intervention did not alter behaviors during non-school days. Furthermore, the CIPRES intervention was primarily effective among highly deprived populations. Further research is needed to explore strategies for extending these positive effects to non-school days and to assess the long-term sustainability of these changes and their impact on broader health outcomes.

## Data Availability

The raw data supporting the conclusions of this article will be made available by the authors, without undue reservation.
